# Investigating Impostorism Among Undergraduate Medical Students at Sultan Qaboos University: A Questionnaire-Based Study

**DOI:** 10.7759/cureus.45752

**Published:** 2023-09-22

**Authors:** Abdullah Al Lawati, Anas Al Wahaibi, Fatma Al Kharusi, Moon Fai Chan, Hamed Al Sinawi

**Affiliations:** 1 College of Medicine and Health Sciences, Sultan Qaboos University, Muscat, OMN; 2 Psychiatry and Behavioral Sciences, Sultan Qaboos University Hospital, Muscat, OMN; 3 Family Medicine and Public Health, Sultan Qaboos University, Muscat, OMN

**Keywords:** prevalence rate, oman, medical school students, college mental health, impostor syndrome

## Abstract

Background

Imposter syndrome or phenomenon (IP) is a behavioral phenomenon observed in successful individuals where they fail to recognize and internalize their achievements. It is often accompanied by feelings of self-doubt, anxiety, depression, and worries of being exposed as frauds, with the impostors often attributing their achievements to external factors like good luck and timing. The presence of IP among medical students is gaining more attention, with studies reporting a strong association with burnout phenomenon, anxiety, and depression.

Objectives

This study sought to determine the prevalence of IP among Omani medical students and classify the levels of severity among the sample.

Methodology

This was a cross-sectional, observational study conducted at a public university in Muscat, Oman. The Clance Impostor Phenomenon Scale (CIPS), a validated publicly available questionnaire, was used to determine whether students exhibited impostorism. Students who scored 63 or higher in the CIPS were classified as impostors. In addition, students were also classified based on the severity of their impostorism. As per the CIPS, scores between 41 and 60 indicate mild impostorism, scores between 61 and 80 indicate moderate impostorism, and finally scores between 81 and 100 indicate severe impostorism.

Results

A total of 276 students participated (M 34%, F 66%), of which 144 (52.2%) were found to have IP with 12.7% exhibiting severe impostorism.

Conclusion

The results show that IP is present in significant frequencies among medical students; further studies are needed to address this problem.

## Introduction

The medical school environment is often competitive, as students' abilities, performance, and academic competence are constantly assessed. This may lead to anxiety among the medical student, self-doubt, and the feeling of being an outsider. The feeling of being an outsider or "an impostor" describes the imposter phenomenon (IP). Imposter syndrome or phenomenon is a behavioral phenomenon observed in successful individuals where they fail to recognize and internalize their achievements and is often accompanied by feelings of self-doubt, anxiety, depression, and worries of being exposed as frauds, and they may as well attribute these achievements to external factors like good luck and timing [1,2]. The presence of IP among medical students is gaining more attention, with studies reporting a strong association with burnout phenomenon, anxiety, and depression. IP is not recognized as a psychiatric disorder, as it is not listed in the American Psychiatric Association's Diagnostic and Statistical Manual or the International Classification of Diseases, Tenth Revision (ICD10), but rather it is considered a behavioral pattern leading to distress [2,3]. The exhibition of impostor traits has been associated with negative personality traits, anxiety, and depression [1,4-6]. In addition, the distress caused by IP may lead to serious negative effects, including poor physical health, decreased academic performance, and even suicide and it is known to be associated with burnout syndrome [7,8].

Therefore, given the severity of IP and its negative consequences, we seek to determine the prevalence and severity of IP among undergraduate medical students in Oman using the Clance Impostor Phenomenon Scale (CIPS) and then compare our findings with other medical schools in the region. Similar studies have been conducted in the Middle East region; however, to our knowledge, this is the first study to be conducted in Oman. The reported prevalence of IP varies widely among different studies in different countries, ranging from 22.0% to 60.0% of students [7-13]. We hypothesize that IP may be high among medical students in Oman.

This article was previously posted to the Research Square preprint server on June 26, 2023.

## Materials and methods

Study design

This cross-sectional observational study is conducted using a validated publicly available questionnaire to determine whether the participants, medical students in our case, exhibit impostor phenomenon characteristics and, if so, the extent (severity) of their impostorism.

Sample size

Seven hundred sixty-one medical students are enrolled at the College of Medicine and Health Sciences at Sultan Qaboos University (SQU). Therefore, a sample size of 256 is required as per the sample size for the population mean formula (confidence level of 95%). Approximately 350 students were invited to fill out the form, and after the removal of duplicates, 276 responses remained, with a response rate of 78.9%. All participants were sampled randomly. 

Questionnaire and data collection

Medical students from SQU were invited to fill out the questionnaire, which was disseminated through Google Forms. The description of the study, as well as the consent form, was displayed at the beginning of the form. All data will be collected using Microsoft Excel 2018 (Redmond, WA, USA) spreadsheet. The questionnaire included includes the following:

a) Consent

Ensure that the student has read the consent sheet attached and has given the approval to participate in the study.

b) Demographics sheet

The collected students’ socio-demographic data will include gender, age, nationality, and phase/level of study.

c) Clance Impostor Phenomenon Scale (CIPS)

This evaluation tool helps determine whether the participant exhibits IP characteristics and, if so, the severity of it. Statements are put forth, and participants are asked to indicate how much they agree with each statement. Responses in this scale are categorized using a 5-option Likert scale and are as follows: "very true," "often," "sometimes," "rarely," and "not at all true." This scale consists of 20 questions, and the calculated Cronbach's α-coefficient of the scale is 0.92-0.96, which indicates a high degree of consistency. In addition, strong validity has also been reported in studies that have utilized this scale [14,15]. The scoring ranges from 0 to 100. Values from 0 to 40 indicate few impostor characteristics. Values from 41 to 60 indicate moderate IP. Values from 61 to 80 indicate frequent IP experiences and values from 80 and above indicate frequent and intense IP experiences. In addition, a cutoff score of 63 or higher was used to classify individuals as impostors [7,14,16]. 

Statistical analysis

The analyses were conducted using IBM Statistical Package for Social Sciences (SPSS) software (version 27.0; IBM Corp., Armonk, New York, USA). Basic statistics (e.g., mean, standard deviation, frequency, and percentage) were used to describe the demographic data of the study sample. Statistical tests (e.g., χ² test, Fisher's exact test) were used to examine the differences between impostorism and non-impostorism among preclinical and clinical students and differences among males and females, in addition to other factors.

## Results

A total of 276 medical students from three phases of the medical school program participated in this study, as indicated in Table 1. Of them, 60 (21.7%) were in phase I (preclinical), 95 (34.4%) were in phase II (preclinical), and 121 (43.8%) were in phase III (clinical). One hundred eighty-two (64.9%) of the population under study were female and 94 (34.1%) were male. The interquartile range of the population's median age was 17-26 years, with 21 being the median age. The bulk of the population, 98 (35.5%), had a cumulative grade point average (GPA) of 2.50-2.99, while the remaining 59 (21.4%), 87 (31.5), 23 (8.3%), and 5 (1.8%) had GPAs of 3.50-4.00, 3.00-3.49, 2.00-2.49, and <2.00, respectively.

**Table 1:** Characteristics of the study sample (n=276) IP: imposter phenomenon; SD: standard deviation; CIPS: Clance Impostor Phenomenon Scale. IP: CIPS 63-100; non-IP: CIPS 0-62.

Characteristic	n (%)
Gender	
Female	182 (65.9)
Male	94 (34.1)
Age (years), mean±SD	21.3±2.0
Median [range]	21.0 [17.0-26.0]
Study phase	
I (preclinical)	60 (21.7)
II (preclinical)	95 (34.4)
III (clinical)	121 (43.8)
Cumulative grade point average	
<2.00	5 (1.8)
2.00-2.49	23 (8.3)
2.50-2.99	98 (35.5)
3.00-3.49	87 (31.5)
3.50-4.00 (ref)	59 (21.4)
Cohort (year)	
2013	1 (4.0)
2014	7 (2.5)
2015	12 (4.3)
2016	50 (18.1)
2017	28 (10.1)
2018	43 (15.6)
2019	31 (11.2)
2020	68 (24.6)
2021	34 (12.3)
2022	2 (0.7)
Clance Impostor Phenomenon Scale	
≤40	16 (5.8)
41-60	104 (37.7)
61-80	121 (43.8)
81-100	35 (12.7)
IP group	
IP	144 (52.2)
Non-IP	132 (47.8)

The CIPS criteria were used to evaluate the characteristics of the IP, and it was discovered that the population experienced IP to varying degrees of severity, with only 16 (5.8%) experiencing a few of the characteristics while 104 (37.7%) experienced moderate characteristics. Comparatively, 35 (12.7%) had a more frequent and intensive course of the features, and 121 (43.8%) had encountered them more frequently. One hundred forty-four people (52.2% of the population) were classified as belonging to the IP group because they scored 63 or higher on the CIPS, and the remaining 132 people (47.8%) were classified as belonging to the non-IP group because they are going through a less severe course of IP characteristics. 

Table 2 shows that of the 144 students classified as belonging to the IP group, 98 (68.1%) were female and 47 (31.9%) were male. There was no significant statistical difference between males and females with regard to IP. The interquartile range in the IP group varied from 18 to 26, whereas in the non-IP group, it ranged from 17 to 26. The median age in the IP group was the same as in the non-IP group. Most of our students are admitted into higher education institutions when they are 17 years old, which provides a modest hint as to the role that the medical environment may have played in boosting IP characteristics just a year after enrolling in the medical program. When applying tests on the cohorts included, it is observed from the results that in the significantly younger and older cohorts 2013-2015 and 2020-2022, more students were included in the non-IP group, while the opposite was observed in the 2016, 2017, 2018, and 2019 cohorts as a higher number of these cohorts' students were identified as IP rather than non-IP. Consequently, this is reflected in the results of the three different phases of the medical school as it showed that in the younger population, like phase I, fewer students, 27, were included in the IP group. At the same time, 33 were identified as non-IP, while on the other hand, in the older population, like in phase II, more students, 50, were identified as IP and 45 as non-IP, while in phase III, the majority of the included students, 67, were identified as IP and the rest, 54, were non-IP. No statistically significant difference was observed between any of the groups. 

**Table 2:** Univariate analysis of the imposter phenomenon status of students in demographic factors IP: imposter phenomenon; SD: standard deviation; CIPS: Clance Impostor Phenomenon Scale. Test, chi-square test; IP: CIPS 63-100, non-IP: CIPS 0-62. ^a^Independent t-test.

Variables	IP, n (%)	Non-IP, n (%)	Test (p-value)
	n=144 (52.2%)	n=132 (47.8%)	
Gender			
Female	98 (68.1)	84 (63.6)	0.59 (0.439)
Male	46 (31.9)	48 (36.4)	
Age (years), mean±SD	21.3±1.9	21.2±2.0	0.33^a^ (0.742)
Median [range]	21.0 [18.0-26.0]	21.0 [17.0-26.0]	
Study phase			
I	27 (18.8)	33 (25.0)	1.74 (0.419)
II	50 (34.7)	45 (34.1)	
III (clinical)	67 (46.5)	54 (40.9)	
Cumulative grade point average			
<2.00	2 (1.4)	3 (2.3)	1.47 (0.832)
2.00-2.49	13 (9.2)	10 (7.6)	
2.50-2.99	47 (33.3)	51 (38.9)	
3.00-3.49	48 (34.0)	39 (29.8)	
3.50-4.00	31 (22.0)	28 (21.4)	
Cohort (year)			
2013-2015	8 (5.6)	12 (9.2)	8.50 (0.204)
2016	30 (20.8)	20 (15.2)	
2017	15 (10.4)	13 (9.8)	
2018	24 (16.7)	19 (14.4)	
2019	21 (14.6)	10 (7.6)	
2020	29 (20.1)	39 (29.5)	
2021-2022	17 (11.8)	19 (14.4)	

## Discussion

In our study, the prevalence of IP among medical students at Sultan Qaboos University College of Medicine and Health Sciences is 52%, keeping up with the rates reported elsewhere in the Arabian Peninsula. Prior published studies support the results of our study. According to a study conducted in Saudi Arabia, approximately 58% of young adults are found to have IP [17]. A cross-sectional descriptive study conducted in the Bahrain campus of the Royal College of Surgeons in Ireland (RCSI) shows that 45% of medical students are labeled to have IP [12]. Our study shows a high prevalence of IP, which is consistent with the previous literature reviews in the United States [18]. However, even with a relatively high prevalence of IP in our study, only 13% have a more frequent and intense course of the characteristics based on the CIPS, which is lower in comparison with the study conducted in the Bahrain Campus of RCSI. This finding is possible because they studied the relationship between self-esteem and IP in which they found that the CIPS score is higher among students with low self-esteem [12]. A study done at Dickinson University School of Pharmacy & Health Sciences, New Jersey, among pharmacy residents, shows that more than half of the cohort have a CIPS score of ≥62. They correlated this finding with long work hours, low self-esteem, and depression [19].

In the present study, it was clearly shown that almost 68% of the IP group is female. However, the p-value is insignificant, which can be explained by several reasons. First, 66% of responses are from females because our college's male-to-female ratio is 3:1. Second, in our society, females tend to have more support from family and friends [20,21]. Third, the number of married female medical students is more compared to male students, and marriage was associated with less IP as it works as a buffer [12]. Corresponding findings are reported from Saudi Arabia, in which 64% of the IP group are females. However, it is statistically significant because nearly a similar number of male-to-female ratio was involved in their study [17]. On the other hand, a study in Bahrain shows no significant gender differences in IP [12]. Previous literature conducted among American medical students shows that the female gender is significantly associated with IP, with more than double the percentage of females displaying IP than their male counterparts [18]. The significance of gender implications and its association in the development of IP is explained to be more in high-achieving women due to the introjection of social gender-role stereotyping and particular family dynamics, despite academic and professional accomplishments [22].

We studied the correlation between cumulative GPA and the presence of impostorism, which is statistically insignificant, in which more than two-thirds of students from the IP group are above 2.5, the cut-point grade in the college curriculum system. As in our curriculum system, students are classified into three phases. As explained earlier, almost 47% of the IP group is from phase III/clinical years, followed by phase II/transitional preclinical years with 35%. This factor is statistically not significant. It corresponds with the study in Bahrain that shows that more than half of the students are from clinical years, and found no significant differences in CIPS between the years of study, including the critical transitional year [12].

It is essential to highlight that major transitions are crucial periods when IP is likely to occur. Particularly in phase II (classroom to the clinic) transition, the present study finds higher IP in phase II compared to phase I. Nonetheless, clinical years are higher due to graduation stress and increased responsibilities.

The perceived fraudulence, fraud syndrome, imposter experience, and imposter phenomenon are all synonyms of imposter syndrome, which was described at first in high-achieving women in 1978 by Clance and Imes [1]. It is generally known as a behavioral health phenomenon when individuals experience extreme self-doubt and fail to acknowledge their success. It usually attributes it to external factors like good timing or luck. Subsequently, individuals experience anxiety, depression, and fears of being exposed to fraudulence despite their success [23]. Since Clance and Imes's original identification of the IP, its criteria have expanded significantly. There is a known relationship between IP and other behavioral health disorders, including anxiety, burnout, depression, and exacerbation of other behavioral health issues [24].

In a highly competitive and challenging environment like medical school, several mental and behavioral issues find a well-nourished ground to develop and grow. Evidence suggests that medicine carries a certain risk to the mental health of medical physicians and students [25]. One of the global meta-analysis studies shows an overall prevalence of depressive symptoms and suicidal ideation in medical students to be 27.2% and 11.1%, respectively [24]. On the other hand, a recent global meta-analysis suggests that anxiety is most prevalent among Middle Eastern and Asian medical students [26]. In 2017, a cross-sectional study was conducted among 662 medical students at SQU; the results show a 7.4% prevalence of burnout syndrome and 24.5% depressive symptoms [27]. Along with the anxiety and depression medical students are at risk of, their perception of the medical college as a place where they are continuously scrutinized can build up feelings of intellectual fraudulence and phoniness [12].

After the first identification of IP in high-achieving women by Clance, and with the increased interest in the phenomena, arguments emerged on the reliability of the IP measurement by Clance, as it initially measured the presence of IP in females. As a result, further measurement instruments are established as several research studies show that men and women experience IP at similar rates [14]. Among the scales used to assess IP is Harvey's scale, a list of 14 statements scored 1-7. The total scores range from 0 to 84, where higher scores indicate a higher degree of impostorism [28]. As for the one used in this study, it is a new Clance IP scale developed in 1985 from the original one established in 1978 after the criticism of IP measurement raised by the researchers [14]. This scale is a 20-item instrument that assesses some clinical attributes and feelings not addressed by Harvey's imposter scale. It is as well positively worded to minimize social desirability effects [29]. According to studies, the Clance IP scale is more sensitive and has reduced the incidence of false positives and false negatives [14]. With all these contributing factors, it was a better option for our study. 

Given the nature of this study, there are some limitations that future studies should aim to minimize. First, the study itself is based on a self-administered questionnaire, and thus there could be a potential for bias, including social desirability bias. Another limitation is the language of the questionnaire. The questionnaire was entirely in English, and while the curriculum at SQU is taught in English, the language might still be a barrier to the participants' complete understanding of the questions. Finally, this study was conducted at SQU only, and thus the findings cannot be generalized for all medical students in Oman as it is a single-center study.

## Conclusions

IP is commonly observed among medical students and can negatively impact their physical and mental well-being and their personal, academic, and professional growth. It is crucial to identify and address IP among students and provide training for both students and teachers.
